# Analgesic and Anti-Inflammatory Activities of Methanol Extract of *Cissus repens* in Mice

**DOI:** 10.1155/2012/135379

**Published:** 2012-09-06

**Authors:** Ching-Wen Chang, Wen-Te Chang, Jung-Chun Liao, Yung-Jia Chiu, Ming-Tsuen Hsieh, Wen-Huang Peng, Yu-Chin Lin

**Affiliations:** ^1^Department of Chinese Pharmaceutical Sciences and Chinese Medicine Resources, College of Pharmacy, China Medical University, 91 Hsueh-Shih Road, Taichung 404, Taiwan; ^2^Department of Pharmacy, College of Pharmacy, China Medical University, Taichung 404, Taiwan; ^3^Department of Biotechnology, TransWorld University, 1221 Zhennan Rd., Douliu City, Yunlin County 640, Taiwan

## Abstract

The aim of this study was to investigate possible analgesic and anti-inflammatory mechanisms of the CR_MeOH_. Analgesic effect was evaluated in two models including acetic acid-induced writhing response and formalin-induced paw licking. The anti-inflammatory effect was evaluated by **λ**-carrageenan-induced mouse paw edema and histopathologic analyses. The results showed that CR_MeOH_ (500 mg/kg) decreased writhing response in the acetic acid assay and licking time in the formalin test. CR_MeOH_ (100 and 500 mg/kg) significantly decreased edema paw volume at 4th to 5th hours after **λ**-carrageenan had been injected. Histopathologically, CR_MeOH_ abated the level of tissue destruction and swelling of the edema paws. These results were indicated that anti-inflammatory mechanism of CR_MeOH_ may be due to declined levels of NO and MDA in the edema paw through increasing the activities of SOD, GPx, and GRd in the liver. Additionally, CR_MeOH_ also decreased IL-1**β**, IL-6, NF**κ**B, TNF-**α**, COX-2, and iNOS levels. The contents of two active ingredients, ursolic acid and lupeol, were quantitatively determined. This paper demonstrated possible mechanisms for the analgesic and anti-inflammatory effects of CR_MeOH_ and provided evidence for the classical treatment of *Cissus repens* in inflammatory diseases.

## 1. Introduction

Inflammatory reaction, typically characterized by redness, swelling, heat and pain, is one of the most important host defense mechanisms against invading pathogens. However, persistent or overinflammation leads to tissue damage and possibly failure of organs. Proinflammatory cytokines (e.g., TNF-*α*, IL-6, and IL-1*β*) are produced in large quantities by activated macrophages/monocytes that stimulate cellular responses via increasing prostaglandins (PGs) and reactive oxygen species (ROS). Additionally, lipid peroxidation (malondialdehyde, MDA) is produced by free radicals attacking the cell membranes. Thus, inflammatory effect results in the accumulation of MDA [1].

*Cissus repens* Lamk. belongs to the family Vitaceae and is distributed in India to southern China, the Philippines, Malaysia, and Taiwan. Several studies have been performed on the composition of *C. repen*, and a number of compounds have been identified such as ursolic acid, asiatic acid, lupeol, friedilin, and epifriedelanol [2]. The roots and stems of *C. repens* are used for snake bites, rheumatic pain, and carbuncles in folk medicine, and the stems are also applied to the treatment of nephritis, long-term coughs, and diarrhoea [3]. However, no research has been investigated on the analgesic and anti-inflammatory mechanisms of *C. repens* yet. 

Many studies have indicated that flavonoids in herbs possess anti-inflammatory activities via scavenging ROS and reducing proinflammatory cytokines (e.g., NF-*κ*B, TNF-*α*, IL-1*β*, and IL-6), such as ursolic acid [4–6] and lupeol [7]. These two ingredients have also been isolated from *C. repens* in previous studies [2, 8]. In this study, not only did we reconfirm the presence of these three compounds in CR_MeOH_ by establishing its fingerprint chromatogram, but the contents of these two active ingredients were quantitatively determined as well.

In this study, we investigated the analgesic and anti-inflammatory activities of the methanol extract of *C. repens* (CR_MeOH_). The analgesic activity was evaluated by acetic acid-induced writhing response and formalin test. Anti-inflammatory activity was determined by using *λ*-carrageenan-induced mouse paw edema model and histopathologic analysis. In order to evaluate the mechanism of anti-inflammatory effect, we also analyzed the levels of TNF-*α*, IL-1*β*, IL-6, NF*κ*B, COX, MDA, and NO in the edema tissues, as well as the activities of superoxidase dismutase (SOD), glutathione peroxidase (GPx), and glutathione reductase (GRd) in the liver.

## 2. Materials and Methods

### 2.1. Chemicals and Drugs

*λ*-carrageenan, indomethacin, and Griess reagent were purchased from Sigma-Aldrich Chemical Co. (Missouri, USA). Formalin was purchased from Nihon Shiyaku Industry Ltd. (Taipei, Taiwan). Murine IL-1*β*, IL-6, and TNF-*α* enzyme-link immunosorbent assay (ELISA) Development Kit was purchased from BioLegend, Inc (California, USA). The SOD, GPx, GRd, COX, TNF-*α*, and NF-*κ*B enzyme-link immunosorbent assay (ELISA) Development Kits were purchased from Cayman Chemical Company (Michigan, USA). Anti-iNOS antibody was purchased from Santa Cruz (rabbit polyclonal to iNOS, sc-650, California, USA). Indomethacin was suspended in 0.5% (w/v) carboxymethylcellulose sodium (CMC) and administered intraperitoneally to animals. LC-grade acetonitrile was purchased from Merck (Darmstadt, Germany). All other reagents used were of analytical grade.

### 2.2. Plant Material

*C. repens* was collected from Yunlin Township of Taiwan and was identified by Dr. Yu-Chin Lin, Leader of the School of Sciences and TransWorld University Department of Biotechnology (TWUBIOT). The voucher specimen (Number: TWU-BIOT-PTE-10101) was deposited at TWUBIOT. A plant specimen has been deposited in the School of Chinese Pharmaceutical Sciences and Chinese Medicine Resources. Dried root (1.0 kg) of *C. repens *was sliced into small pieces and extracted under reflux with 10 liters of methanol three times. The combined extract was concentrated under reduced pressure to give a dried extract (yield ratio 7.87%). The dried crude extract was dissolved in 0.5% CMC solution prior to pharmacological tests.

### 2.3. Chromatographic Analysis of CR_MeOH_

The HPLC system consisted of a Shimadzu (Kyoto, Japan) LC-10ATvp liquid chromatograph equipped with a DGU-14A degasser, an FCV-10ALvp low-pressure gradient flow control valve, an SIL-10ADvp autoinjector, an SPD-M10Avp diode array detector, and an SCL-10Avp system controller. Peak areas were calculated using Shimadzu Class-LC10 software (Version 6.12 sp5). The column was a Vercopak Inertsil 7 ODS-3 (5 *μ*m, 4.6 × 150 mm) Vercotech Inc. column. The mobile phase consisted of a mixture of 0.2% phosphoric acid (A) and acetonitrile (B) using a gradient elution. The sample was injected of 10 *μ*L. The following gradient profile was run at 1.0 mL/min over 60 min. Ursolic acid gradient program was set as follows: 0–25 min, 25% B, 25–55 min, 90% B, 55–60 min, 25% B. Peaks were detected at 304 nm with SPD-M10AVP (Shimadzu) detector. Lupeol gradient program was set 0–40 min, solvent A : B = 5 : 95. Peaks were detected at 205 nm with SPD-M10AVP (Shimadzu) detector. The above conditions were used in both HPLC assay and HPLC fingerprint of CR_MeOH_. 

The contents of ursolic acid and lupeol of CR_MeOH_ were qualified and quantified in the HPLC assay. In qualitative analysis, comparisons were made with the retention time and maximum absorption of the standards. In quantitative analyses, comparisons were made with peak areas under the standard curves.

### 2.4. Experimental Animals

 Male ICR mice (20–25 g) were obtained from BioLASCO Taiwan Co., Ltd. The mice were kept in the animal center of China Medical University at a controlled temperature of 22 ± 1°C, relative humidity 55 ± 5*%*, and with 12 h light/12 h dark cycles for 1 week before the experiment. Animals were provided with rodent diet and clean water *ad libitum*. All studies were conducted in accordance with the National Institutes of Health (NIH) Guide for the Care and Use of Laboratory Animals. All tests were conducted under the guidelines of the International Association for the Study of Pain [9]. The experimental protocol was approved by the Committee on Animal Research, China Medical University. This study used cervical dislocation to sacrifice animal.

### 2.5. Acute Toxicity Study

The acute toxicology test in mice was carried out according to the method of Liao et al. [10]. Male ICR mice were random divided into three groups (10 mice per group). The mice were administered orally with CR_MeOH_ (2.5 g, 5 g, and 10 g/kg). The experimental mice were provided with forage and water ad libitum, and they were kept under regular observation for 14 days for any mortality or behavioral changes. The behavioral changes closely observed for were hyperactivity, tremors, ataxia, convulsions, salivation, diarrhea, lethargy, sleep, and coma.

### 2.6. Acetic-Acid-Induced Writhing Response

The writhing test in mice was carried out according to the method of Koster et al. [11]. The writhes were induced by intraperitoneal injection of 1.0% acetic acid (v/v, 0.1 ml/10 g body weight). There are three different doses (20, 100, and 500 mg/kg) of CR_MeOH_ administered orally to each groups of mice, 60 min before chemical stimulus. Indomethacin as a positive control was administered 30 min prior to acetic acid injection. The number of muscular contractions was counted over a period of 5 min after acetic acid injection. The data represented the total numbers of writhes observed during 10 min.

### 2.7. Formalin Test

The formalin test was conducted based on the method of Tjølsen et al. [12]. Twenty microliter of 5% formalin in saline was injected subcutaneously into the right hind paw of each mouse. The time (in seconds) spent in licking and biting responses of the injected paw was taken as an indicator of pain response. Responses were measured for 5 min after formalin injection (early phase) and 20–30 min after formalin injection (late phase). CR_MeOH_ (20, 100, and 500 mg/kg, p.o.) was administered 60 min before the formalin injection. Indomethacin (10 mg/kg, i.p.) was administered 30 min before formalin injection.

### 2.8. *λ*-Carrageenan-Induced Mouse Paw Edema

The anti-inflammatory activity of CR_MeOH_ was determined by the *λ*-carrageenan-induced paw edema test in the hind paws of mice. The test was conducted according to the method of Vinegar et al. [13]. The basal volume of right hind paw was determined before the administration of any drug. Fifty microliter of 1%  *λ*-carrageenan suspended in saline was injected into the plantar side of right hind paw, and the paw volume was measured at the 1st, 2nd, 3rd, 4th, and 5th hours after the injection using an MK101 CMP plethysmometer (Muromachi Kikai Co., Ltd). The degree of swelling was evaluated by the delta volume (a-b), where “a” is the volume of right hind paw after the chemical treatment and “b” is the volume before the treatment. Indomethacin (10 mg/kg) was administered intraperitoneally 30 min before *λ*-carrageenan injection. CR_MeOH_ (20, 100, and 500 mg/kg) was orally administered 60 min before *λ*-carrageenan injection. The control was given an equal volume of saline. 

In the secondary experiment, another set of mice were orally administered with 0.5% CMC, indomethacin or CR_MeOH_ 1 h before *λ*-carrageenan had been injected into their right hind paws. The right hind paws of the animals were taken 4 h later. The paw tissue was rinsed in ice-cold normal saline and immediately placed in cold normal saline four times its volume before homogenization at 4°C. Then, the homogenate was centrifuged at 12,000 rpm for 5 min. The supernatant was obtained and stored at −20°C for upcoming MDA, NO, TNF-*α*, IL-1*β*, IL-6, NF*κ*B, and COX analysis. As for the whole liver tissue, similarly, it was rinsed in ice-cold normal saline and immediately placed in cold normal saline of equal volume before homogenization at 4°C. The homogenate was then centrifuged at 12,000 rpm for 5 min. The supernatant was obtained and stored at −20°C for later analysis of antioxidant enzymes (SOD, GPx, and GRd) activities.

### 2.9. Histological Analysis

For histopathological examination, biopsies of paws were taken 4 h after the induction with carrageenan. Tissue slices were fixed in 10% formalin for 3 days, decalcified overnight, embedded in paraffin, and sectioned into 4 *μ*m tissue sections. Tissue sections were stained with hematoxylin and eosin (H&E stain) and examined with a BX60 microscope (Olympus, Melville, NY) for pathological changes. Inflammatory reactions induced by *λ*-carrageenan, including paw swelling, were examined. The enlarged cavities after CR_MeOH_ (20 mg, 100 mg, and 500 mg/kg) and indomethacin (10 mg/kg) treatments were also examined. Images were captured with a Macrofire 599831 camera. The results were identified in the Animal Disease Diagnostic Center (ADDC), National Chung Hsing University, Taichuang, Taiwan.

### 2.10. MDA Assay

The production of MDA was induced by *λ*-carrageenan injection and evaluated by the thiobarbituric acid reacting substance (TBARS) method [14]. In brief, MDA reacted with TBARS at high temperature and formed a red-complex TBARS. The absorbance of TBARS was recorded at 532 nm.

### 2.11. NO Assay

NO was measured according to the method of Moshage et al. [15]. NO_3_^−^ was converted into NO_2_^−^ by nitrate reductase, NO_2_^−^ subsequently reacted with sulfanilic acid to produce diazonium ion and coupled with N-(1-naphthyl) ethylenediamine to form the chromophoric azo-derivative (purplish red) which could be recorded at 540 nm.

### 2.12. TNF-*α*, IL-1*β*, IL-6, and NF*κ*B Assay

IL-1*β* was measured by an enzyme-linked immunosorbent assay [16]. The capture antibody of IL-1*β* was seeded to each well of a 96-well plate overnight. Next day, a second set of biotinylated antibody was incubated with sample tissues or standard antigens in the plate before streptavidin was finally added. The color of the reaction converted from purple to yellow and was recorded at 450 nm. IL-6, TNF-*α*, and NF*κ*B were detected using the same method as IL-1*β*. Each sample was presented as ng/mg in TNF-*α*, IL-6, IL-1*β*, and NF*κ*B concentrations.

### 2.13. COX-2 Assay

The content of COX-2 was determined by measuring the peroxidase activity of PGHS (prostaglandin endoperoxide H2 synthase) [17]. Peroxidase activity of PGHS was determined by following the oxidation of N,N,N′,N′-tetramethyl-p-phenylenediamine (TMPD) at 37°C using arachidonate as the substrate. The increase in color was recorded at 590 nm.

### 2.14. Measurement of Antioxidant Enzymes

SOD was measured according to the method of Vani et al. [18]. Xanthine and xanthine oxidase (XOD) generated superoxide radicals reacted with 2-(4-iodophenyl)-3-(4-nitrophenol)-5-phenyl-tetrazolium chloride (I.N.T.) to form a red formazan dye, and the color was recorded at 540 nm. GPx was measured according to the method of Ceballos-Picot et al. by detecting the contents of GR and NADPH [19]. Oxidation of NADPH into NADP^+^ is accompanied by a decrease in absorbance recorded at 340 nm. GRd was measured according to the method of Ahmad and Holdsworth [20] which detects the decrease of glutathione (GSSG) in the presence of NADPH. NADPH oxidized into NADP^+^ would result in a decrease in absorbance recorded at 340 nm.

### 2.15. Western Blotting for iNOS

Freshly isolated paw tissue was homogenized in a lyses buffer. The protein concentration of the tissue homogenate and the cytosolic and microsomal fractions were determined according to the method of Lowry et al. [21]. One hundred *μ*g of protein from paw homogenates or 50 *μ*g of protein from purified microsome were loaded per lane on 8% or 12% polyacrylamide gels and electrophoresis was performed. Proteins were then transferred onto nitrocellulose membranes. The membrane was blocked overnight with buffer and then incubated with primary antibodies for 1 h using 1 : 1000 dilution of rabbit anti-iNOS (100-fold dilution, 2 h at 25°C, Santa Cruz). The membranes were then washed three times in TBST solution containing Tris buffer solution (TBS) with 0.1% Tween-20 for 15 min, incubated with 1 : 1000 dilution of alkaline phosphatase-conjugated anti-rabbit IgG as the second antibody for 1 h. The protein was visualized with an enhanced chemiluminescence Western blotting detection kit (Amersham, Arlington Heights, IL, USA), and exposed to X-ray film for 3 min.

### 2.16. Statistical Analysis

 All data represented the mean ± SEM. Statistical analyses were performed with SPSS software, and were carried out using one-way ANOVA followed by Scheffe's multiple range test.

## 3. Results

### 3.1. Chromatographic Analysis of CR_MeOH_

The HPLC chromatogram shows that ursolic acid and lupeol with retention times of 28.94 min and 29.18 min, respectively. The maximum absorbance was 304 nm, and the relative amounts were in the order of ursolic acid (69.16 mg/g) and lupeol (8.65 mg/g).

### 3.2. Acute Toxicity Study

Acute toxicity of CR_MeOH_ was evaluated in mice at the doses of 2.5, 5, and 10 g/kg. After 14 days of oral administration, CR_MeOH_ did not cause any behavioral changes, and no mortality was observed. Therefore, the LD_50_ value of CR_MeOH_ was concluded to be greater than 10 g/kg in mice, indicating it was practically non acute toxic.

### 3.3. Acetic-Acid-Induced Writhing Response

**Figure 1:**
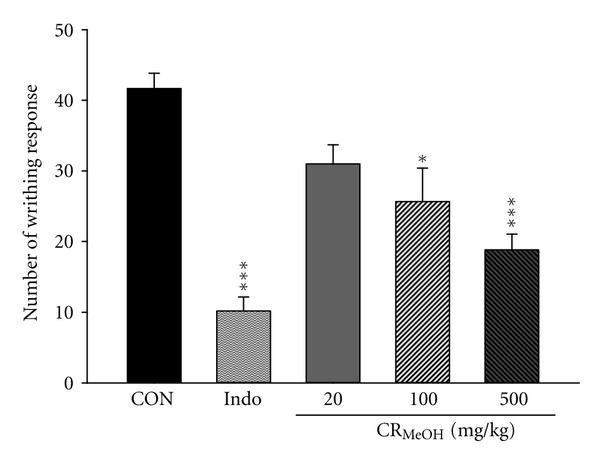
Analgesic effect of CR_MeOH_ and indomethacin (Indo) on acetic-acid-induced writhing response in mice. Each value represents mean ± SEM (*n* = 8). *P < 0.05 and ***P < 0.001 as compared to the solvent control (CON) group (one-way ANOVA followed by Scheffe's multiple range test).

Figure 1 shows acetic acid-induced writhing responses in mice which serve as an indication of analgesic activities of CR_MeOH_. Intraperitoneal injection of acetic acid produced 41.67 ± 5.28 writhes in the solvent control group. The writhing response was significantly reduced by pretreatments with 500 mg/kg CR_MeOH_ (18.83 ± 2.21, *P* < 0.001) and 10 mg/kg indomethacin (10.17 ± 1.99, *P* < 0.001).

### 3.4. Formalin Test

**Figure 2:**
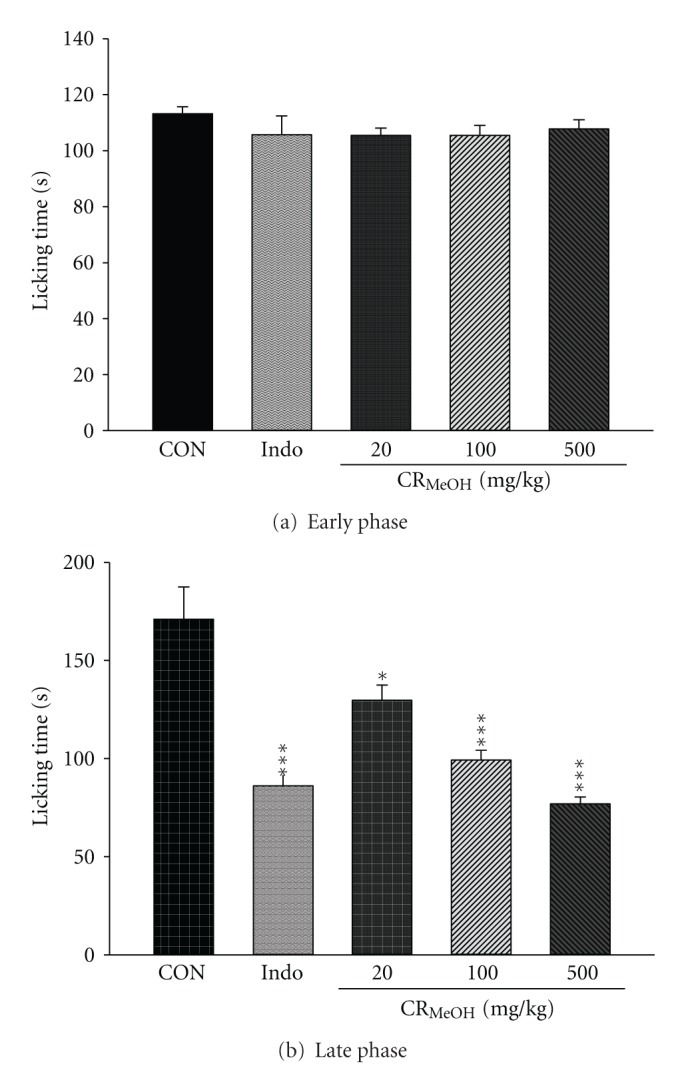
Effect of CR_MeOH_ and indomethacin (Indo) on (a) early phase and (b) late phase of formalin test in mice. Each value represents mean ± SEM (*n* = 8). *P < 0.05 and ***P < 0.001 as compared to the solvent control (CON) group (one-way ANOVA followed by Scheffe's multiple range test).

CR_MeOH_ demonstrated a dose-dependent relationship in late phase of formalin-induced pain. In the early phase, CR_MeOH_ (20, 100, and 500 mg/kg) and indomethacin (10 mg/kg) treated groups did not show any significant changes as compared to the control group (Figure 2(a)). In the late phase, subcutaneous injection of formalin-induced licking and biting responses which lasted a duration of 171.0 ± 16.44 s. The time was significantly decreased by pretreatment with 100 and 500 mg/kg CR_MeOH_ (99.29 ± 4.90, *P* < 0.001 and 76.89 ± 3.47, *P* < 0.001) and 10 mg/kg indomethacin (85.67 ± 2.74, *P* < 0.001), as shown in Figure 2(b).

### 3.5. Effect of CR_MeOH_ on *λ*-Carrageenan-Induced Mouse Paw Edema

**Figure 3:**
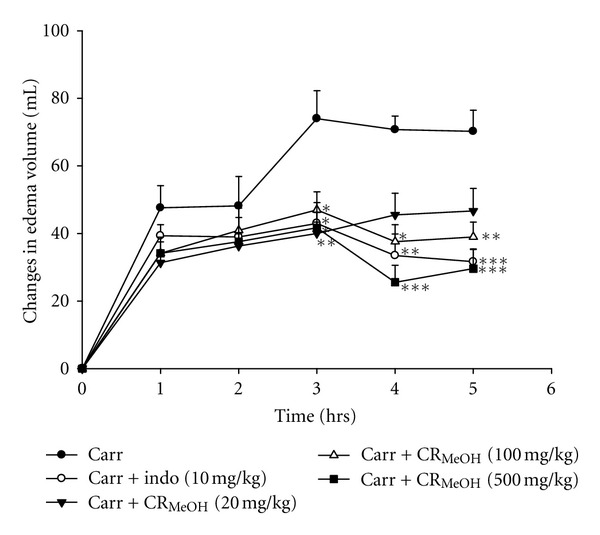
Effect of CR_MeOH_ and indomethacin (Indo) on *λ*-carrageenan induced mouse paw edema. Each value represents mean ± SEM (*n* = 8). *P < 0.05, **P < 0.01, and ***P < 0.001 as compared to the *λ*-carrageenan control group (one-way ANOVA followed by Scheffe's multiple range test).

As shown in Figure 3, after the *λ*-carrageenan injection, the volume of mouse paw increased as edema developed, indicating inflammatory activities. However, indomethacin (10 mg/kg) and CR_MeOH_ (500 mg/kg) obviously decreased paw edema at the 4th and 5th h after the administration. CR_MeOH_ at the dose of 500 mg/kg (29.60 ± 4.40, *P* < 0.001) showed almost equal amount of inhibition as 10 mg/kg indomethacin (31.67 ± 4.40, *P* < 0.001).

### 3.6. Histological Analysis

**Figure 4:**
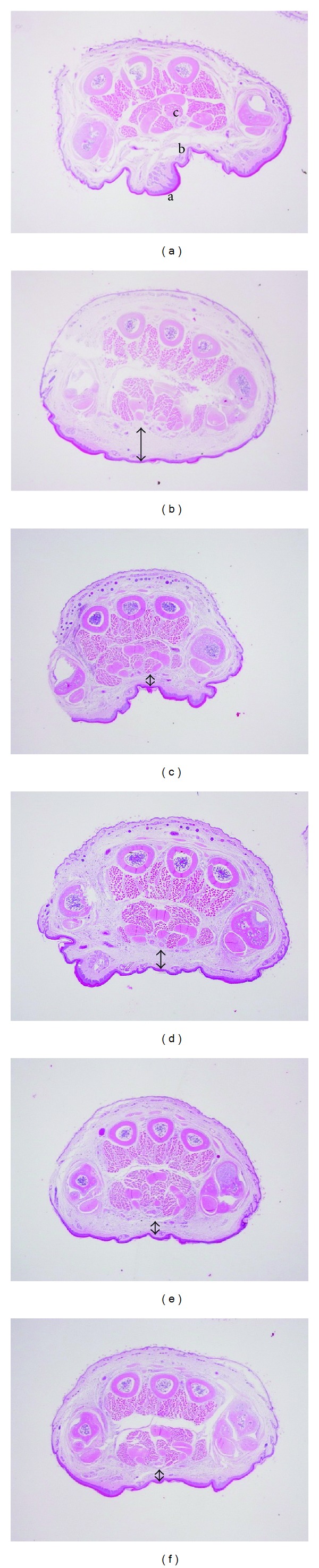
Histopathological examinations on *λ*-carrageenan-induced paw tissue swelling, edema and neutrophil infiltration (a: epidermis, b: connective tissue, c: muscle fibers): (a) normal, (b) *λ*-carrageenan, (c) indomethacin, (d) CR_MeOH_ (20 mg/kg), (e) CR_MeOH_ (100 mg/kg), (f) CR_MeOH_ (500 mg/kg). More swelling of the connective tissue, the distance is greater of the gap.

For histopathological examination, paw biopsies were harvested for 4 h after *λ*-carrageenan had been injected. Both CR_MeOH_ and indomethacin significantly decreased carrageenan-induced paw oedema (Figure 4).  Figure 4(a) shows no inflammation, tissue destruction, and swelling phenomenon in the paws of normal mice. On the other hand, the *λ*-carrageenan group displayed enlarged cavities in the paw tissue (Figure 4(b)). As for the positive and experimental groups, edematous condition was obviously abated by treatment with indomethacin (10 mg/kg) and CR_MeOH_ (20, 100, and 500 mg/kg), as shown in Figures 4(c), 4(d), 4(e), and 4(f).

### 3.7. Effect of CR_MeOH_ on MDA Level

In this study, MDA level was used to signify lipid peroxidation. In Table 1, MDA level dramatically increased in the *λ*-carrageenan group (2.62 ± 0.16 nmol/mg); however, pretreatments with 500 mg/kg CR_MeOH_ (1.274 ± 0.065, *P* < 0.001) and 10 mg/kg indomethacin (1.376 ± 0.096, *P* < 0.001) showed significant inhibition in the increase MDA level. 

### 3.8. Effect of CR_MeOH_ on NO Level

Pretreatments with 100 and 500 mg/kg CR_MeOH_ (6.918 ± 0.318, *P* < 0.001 and 6.222 ± 0.501, *P* < 0.001) and 10 mg/kg indomethacin (6.438 ± 0.321, *P* < 0.001) showed significant inhibition in the increase of NO level in the edema paws of mice, as compared to the *λ*-carrageenan group (9.96 ± 0.44 *μ*M, Table 1).

### 3.9. Effect of CR_MeOH_ on TNF-*α*, IL-1*β*, IL-6, and NF*κ*B

TNF-*α*, IL-1*β*, IL-6, and NF*κ*B levels in *λ*-carrageenan-induced edema paws were remarkably raised. CR_MeOH_ (500 mg/kg) and indomethacin (10 mg/kg) significantly reduced the levels of TNF-*α* (*P* < 0.001). The IL-1*β* and IL-6 levels increased significantly at the 4th h after Carr injection (*P* < 0.01, *P* < 0.001). The IL-1*β*, IL-6, and NF*κ*B levels in serum were significantly decreased by treatment with 500 mg/kg CR_MeOH_ as well as 10 mg/kg indomethacin (Table 1).

### 3.10. Effect of CR_MeOH_ on COX Level

Table 2 shows that COX-2 level was greatly raised (207.273 ± 9.984 U/mg) in *λ*-carrageenan-induced edema paw. However, COX-2 levels were decreased by treating with 500 mg/kg CR_MeOH_ (134.936 ± 9.918, *P* < 0.001) as well as 10 mg/kg indomethacin (135.996 ± 7.489, *P* < 0.001). No such result was found in COX-1 activity assay.

### 3.11. Effect of CR_MeOH_ on the Carr-Induced Mice Paw Edema

**Figure 5:**
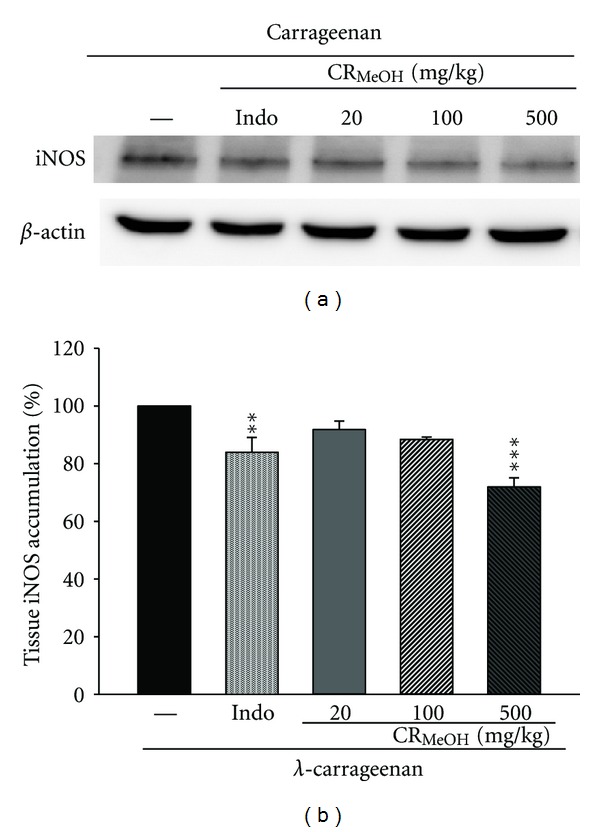
Inhibition of iNOS protein expression by CR_MeOH_ induced by *λ*-carrageenan (Carr) in mice paw edema for 4 h. Tissue suspended were then prepared and subjected to western blotting using an antibody specific for iNOS. *β*-actin was used as an internal control. (a) Representative western blot from two separate experiments is shown. (b) Relative iNOS protein levels were calculated with reference to Carr-injected mouse. The data were presented as mean ± S.D. for three different experiments performed in triplicate. **P < 0.01 and ***P < 0.001 were compared with Carr-alone group.

Results showed that injection of CR_MeOH_ (500 mg/kg) inhibited iNOS (71.9%) proteins expression in Carr-induced (*P* < 0.001) mouse paw edema at the 4th h (Figures 5(a) and 5(b)). However, 10 mg/kg Indomethacin showed an average down-regulation of iNOS (84.0%, *P* < 0.01, Figure 5(b)).

### 3.12. Effect of CR_MeOH_ on the Activities of Antioxidant Enzymes

The antioxidant enzyme activities such as SOD, GPx, and GRd at the 4th hour following the intrapaw injection of *λ*-carrageenan in mice are presented in Table 3. SOD, GPx, and GRd activities in liver tissue were decreased significantly at 4th after *λ*-carrageenan administration. Treatment with CR_MeOH_ (500 mg/kg) and indomethacin (10 mg/kg) increased the SOD, GPx, and GRd activities significantly (*P* < 0.001).

## 4. Discussion

*Cissus* is a genus of about 200 species found in tropical and subtropical regions. In Taiwan, CR is used for the treatment of many diseases, such as epilepsy, stroke, abscess, and diabetes. In addition, it also has anti-inflammatory and antirheumatic activity [22].

Traditional medicines have been popularly used in the treatment of various diseases in recent years. Many medicinal plants supply analgesic and anti-inflammatory activities to treat acute, chronic, or recurring illnesses. Based on its therapeutic claims in traditional medicine, we investigated possible mechanisms of the analgesic and anti-inflammatory effects of CR_MeOH_. 

Analgesic effect of CR_MeOH_ was evaluated by two animal models, including acetic acid-induced writhing response and formalin test. Acetic acid indirectly triggers the release of nociceptive endogenous mediators (such as serotonin and prostaglandin) and proinflammatory cytokines (such as TNF-*α* and IL-1*β*) to cause painful sensation [23]. This nociceptive effect can be prevented by NSAID drugs and analgesic agents with central actions, such as morphine. In the present study, CR_MeOH_ (500 mg/kg) and indomethacin (10 mg/kg) provided antinociceptive effect to relieve abdominal writhes induced by acetic acid in mice.

Formalin test involves a biphasic responses. The first phase (neurogenic nociceptive response) occurs in the first 5 min after the formalin injection. The second phase (inflammatory nociceptive response) occurs between 15 to 30 min after formalin injection. Central acting drugs can inhibit both phases, while peripheral acting drugs, such as NSAID drugs, only inhibit the second phase [24]. The treatments of CR_MeOH_ (100 and 500 mg/kg) and indomethacin (10 mg/kg) were able to diminish the nociceptive response in the second phase induced by the formalin injection. The results indicated that the antinociceptive effect of CR_MeOH_ could be due to its anti-inflammatory effect.

*λ*-Carrageenan-induced paw edema, an *in vivo* model of inflammation, has also been characterized as a biphasic event [13]. Histamine, bradykinin, and 5-hydroxytryptamine (5-HT) are released in the first phase of edema (0-1 h). In the second phase (1–6 h), TNF-*α*, IL-1*β*, COX-2, and PGs are produced more actively, which could in turn worsen the degree of swelling. It is well known that the expression of COX-2 is maximal at the late phase of *λ*-carrageenan-induced paw edema, which could subsequently increase prostaglandin levels in inflammatory reactions [25]. IL-1*β* and TNF-*α* are involved in neutrophil migration in *λ*-carrageenan-induced inflammation. These mediators are able to recruit leukocytes, such as neutrophils, as reported in several recent experimental models [26, 27]. In this study, CR_MeOH_ and indomethacin (10 mg/kg) showed significant anti-inflammatory effect on *λ*-carrageenan induced mouse paw edema from the 4th and 5th. Moreover, the levels of TNF-*α*, IL-1*β*, and IL-6 were also decreased by treating with CR_MeOH_ and indomethacin (10 mg/kg). Thus, a putative anti-inflammatory mechanism of CR_MeOH_ could be associated with the degree of inhibition on inflammatory mediators, such as TNF-*α*, IL-1*β*, and IL-6.

COX-2 selective inhibitor is a form of NSAID that directly targets COX-2, an enzyme responsible for inflammation and pain [28]. Selectivity for COX-2 reduces the risk of peptic ulceration and similar to COX-2, iNOS produces significant amounts of NO and has been identified as playing a central role in inflammatory diseases [29]. Numerous studies have indicated that NO and PGs participate in inflammatory and nociceptive events [30]. Inhibition of NO and PGs production via the inhibition of iNOS and COX-2 expression is beneficial in treating inflammatory diseases [31]. Experimental results indicate that the CR_MeOH_ (500 mg/kg) has an inhibitory effect on iNOS and COX-2 protein expression. The anti-inflammatory mechanism of CR_MeOH_ may be related to the inhibition of PGs and NO synthesis, as described for the anti-inflammatory mechanism of indomethacin in inhibiting the inflammatory process, induced by carrageenan [32].

In histopathological examination of the *λ*-carrageenan group, paw biopsies displayed obvious enlarged cavities in the connective tissue. However, as shown in Figure 5, the indomethacin and CR_MeOH_ groups significantly improved the edematous condition induced by *λ*-carrageenan. The intercellular spaces of connective tissues were also obviously decreased. 

Neutrophil migration, stimulated by *λ*-carrageenan, released active substances such as ROS. Nitric oxide, hydrogen peroxide, hydroxyl radicals, and superoxide anions play major roles in terms of producing cellular damage [33, 34]. MDA is a reactive aldehyde caused by toxic stress in cells and form covalent protein adducts which are referred to as advanced lipid peroxidation end products (ALE). Inflammatory effect would result in the accumulation of MDA. Enhancing the level of glutathione and SOD, on the other hand, could reduce MDA production [35]. In this study, SOD, GPx, and GRd activities increased in the liver after treatment with CR_MeOH_. On the other hand, CR_MeOH_ decreased significantly the MDA level. Therefore, we suspect that the suppression of MDA production was likely associated to the increase in SOD, GPx, and GRd activities. 

The fingerprint chromatogram of CR_MeOH_ was established. Its two triterpenoid (ursolic acid and lupeol) contents were quantitatively determined. Two active compounds of CR_MeOH_, ursolic acid and lupeol, have been previously demonstrated to possess anti-inflammatory and antinociceptive activities [36, 37].

This study demonstrated that *Cissus repens* exhibited antinociceptive and anti-inflammatory activities. The anti-inflammatory mechanisms of CR_MeOH_ against *λ*-carrageenan induced paw edema involved two possible pathways. The first pathway was likely associated to the decrease in MDA and NO levels in the edema paw via increasing the activities of SOD, GPx, and GRd in the liver. The other pathway alleviated the levels of inflammatory factors, such as IL-1*β*, IL-6, TNF-*α*, NF*κ*B, iNOS, and COX-2 in the edema paw induced by *λ*-carrageenan. This supported possible mechanisms of CR_MeOH_ used for alleviating inflammatory pain. Moreover, this study also provides the first evidence for its analgesic and inflammation effect in *Cissus repens*.

## Figures and Tables

**Table 1:** Effect of CR_MeOH_ and indomethacin on MDA, NO, TNF-*α*, IL-1*β*, IL-6, and NF*κ*B level in the edema paws.

Groups	MDA (nmol/mg protein)	NO (*μ*M)	TNF-*α* (pg/mg protein)	IL-1*β* (ng/mg protein)	IL-6 (ng/mg protein)	NF*κ*B (ng/mg protein)
Carr	2.619 ± 0.163	9.956 ± 0.437	297.600 ± 15.539	929.167 ± 60.585	1483.900 ± 80.219	0.842 ± 0.088
Carr + Indomethacin	1.376 ± 0.096***	6.438 ± 0.321***	190.000 ± 11.779***	639.500 ± 14.040*	1113.500 ± 74.330*	0.527 ± 0.032**
Carr + CR_MeOH _(20 mg/kg)	2.328 ± 0.149	7.699 ± 0.398*	250.000 ± 16.441	922.000 ± 76.033	1239.000 ± 70.825	0.538 ± 0.039**
Carr + CR_MeOH _(100 mg/kg)	2.001 ± 0.187*	6.918 ± 0.318***	212.000 ± 8.569**	740.000 ± 35.407	1022.400 ± 89.513**	0.516 ± 0.042***
Carr + CR_MeOH _(500 mg/kg)	1.274 ± 0.065***	6.222 ± 0.501***	191.917 ± 9.339***	606.000 ± 50.212**	978.444 ± 44.539***	0.493 ± 0.014***

Each value represents the mean ± S.E.M. (*n* = 8). *P < 0.05, **P < 0.01, and ***P < 0.001 as compared with the *λ*-carrageenan (Carr) group (one-way ANOVA followed by Scheffe's multiple range test).

**Table 2:** Effect of CR_MeOH_ and indomethacin on COX concentrations in mouse paw edema.

Groups	COX-1 (U/mL/mg protein)	COX-2 (U/mL/mg protein)
Carr	133.588 ± 5.583	207.273 ± 9.984
Carr + Indomethacin	90.850 ± 2.963***	135.669 ±7.489***
Carr + CR_MeOH _(20 mg/kg)	133.188 ± 6.656	162.505 ± 12.750
Carr + CR_MeOH _(100 mg/kg)	132.383 ± 7.511	148.923 ± 14.681*
Carr + CR_MeOH _(500 mg/kg)	129.140 ± 5.214	134.936 ± 9.918***

Each value represents the mean ± S.E.M. (*n* = 8). *P < 0.05 and ***P < 0.001 as compared with the *λ*-carrageenan (Carr) group (one-way ANOVA followed by Scheffe's multiple range test).

**Table 3:** Effect of CR_MeOH_ and indomethacin on the liver SOD, GPx, and GRd activities in mice injected with **λ**-carrageenan.

Groups	SOD (U/mg protein)	GPx (U/mg protein)	GRd (U/mg protein)
Carr	4.42 ± 0.21	0.066 ± 0.005	0.217 ± 0.033
Carr + Indomethacin	6.43 ± 0.27***	0.179 ± 0.028*	1.215 ± 0.049***
Carr + CR_MeOH _(20 mg/kg)	5.31 ± 0.24	0.139 ± 0.024	0.892 ± 0.098**
Carr + CR_MeOH _(100 mg/kg)	5.42 ± 0.23	0.230 ± 0.028**	1.260 ± 0.095***
Carr + CR_MeOH _(500 mg/kg)	6.24 ± 0.17 ***	0.255 ± 0.022***	1.658 ± 0.035***

Each value represents the mean ± S.E.M. (*n* = 8). *P < 0.05, **P < 0.01, and ***P < 0.001 as compared with the *λ*-carrageenan (Carr) group (one-way ANOVA followed by Scheffe's multiple range test).
